# The effect of polydeoxyribonucleotide prolotherapy on posterior tibial tendon dysfunction after ankle syndesmotic surgery: A case report

**DOI:** 10.1097/MD.0000000000005346

**Published:** 2016-12-23

**Authors:** Tae-Ha Lim, Hyung Rae Cho, Keum Nae Kang, Chang Joon Rhyu, Sung Won Chon, Young Su Lim, Jee In Yoo, Jung-Won Kim, Young Uk Kim

**Affiliations:** a Department of Anesthesiology and Pain Medicine, Eulji General Hospital, Eulji University College of Medicine, Seoul; b Department of Anesthesiology and Pain Medicine, Myongji Hospital, College of Medicine, Seonam University, Goyang; c Department of Anesthesiology and Pain Medicine, National Police Hospital, Seoul; d Department of Anesthesiology and Pain Medicine, Institute for Integrative medicine, Catholic Kwandong University of Korea College of Medicine, International ST. Mary's Hospital, Incheon, Republic of Korea.

**Keywords:** ankle syndesmotic surgery, case report, polydeoxyribonucleotide, posterior tibial tendon dysfunction, prolotherapy

## Abstract

**Rationale:**

Ankle syndesmotic injuries occasionally require long-term therapy for recovery and can result in tendon injury. Posterior tibial tendon dysfunction (PTTD) is an acquired deformity that can cause flatfoot deformity. The current nonoperative management of PTTD includes nonsteroidal antiinflammatory drugs (NSAIDs), orthopedic devices. Although various treatment options have been attempted, optimal treatments for each stage of the condition are debated. Polydeoxyribonucleotide (PDRN) is effective in healing of chronic wounds associated with tissue damage by attracting tissue growth factors.

**Patient concerns:**

A 67-year-old woman who presented at our pain clinic with pain on the inside of ankle. She had a syndesmotic screw fixation 3 years prior. Her ankle pain had persisted after the removal of screws and edema for about 1 month resulting from long-term NSAIDs administration.

**Diagnoses:**

The origin of the pain was possibly tibialis posterior muscle and posterior tibial tendon and she was diagnosed as PTTD after syndesmosis surgery.

**Interventions:**

Sono guided prolotherapy with PDRN was carried out.

**Outcomes:**

Patient showed improvement in the arch of the foot, experienced pain relief, and was able to wear regular shoes without any orthopedic device.

**Lessons:**

This case report highlights that PDRN prolotherapy is a safe and efficient therapeutic option for the treatment of PTTD.

## 1 Introduction

Ankle syndesmotic injuries occur in more than 10% of fractures.^[1]^ Surgical management with syndesmosis screw fixation has been performed successfully and is considered the standard of operation.^[1]^ However, syndesmosis injuries occasionally contribute to chronic instability and tendon injury.^[2]^ Posterior tibial tendon dysfunction (PTTD) is an acquired deformity that can result in the development of painful flatfoot deformity.^[3,4]^ Conservative management measures include rehabilitation program, nonsteroidal antiinflammatory drugs (NSAIDs), and physical therapy.^[5]^ Although various therapeutic options have been attempted, the optimal treatment strategy for the PTTD is unclear.^[4]^ Polydeoxyribonucleotide (PDRN, Placentex integro; Mastelli srl, San Remo, Italy) promotes healing in chronic wounds involving tissue damage by stimulating tissue reconstruction without any adverse effects.^[6]^ Prolotherapy is the injection of an irritant into a tendon insertion or ligament site with the main purpose of pain relief.^[7]^ Herein, we reported a patient who received prolotherapy with PDRN for the maintenance and treatment of PTTD after ankle syndesmotic surgery.

## 2 Case presentation

**Figure 1:**
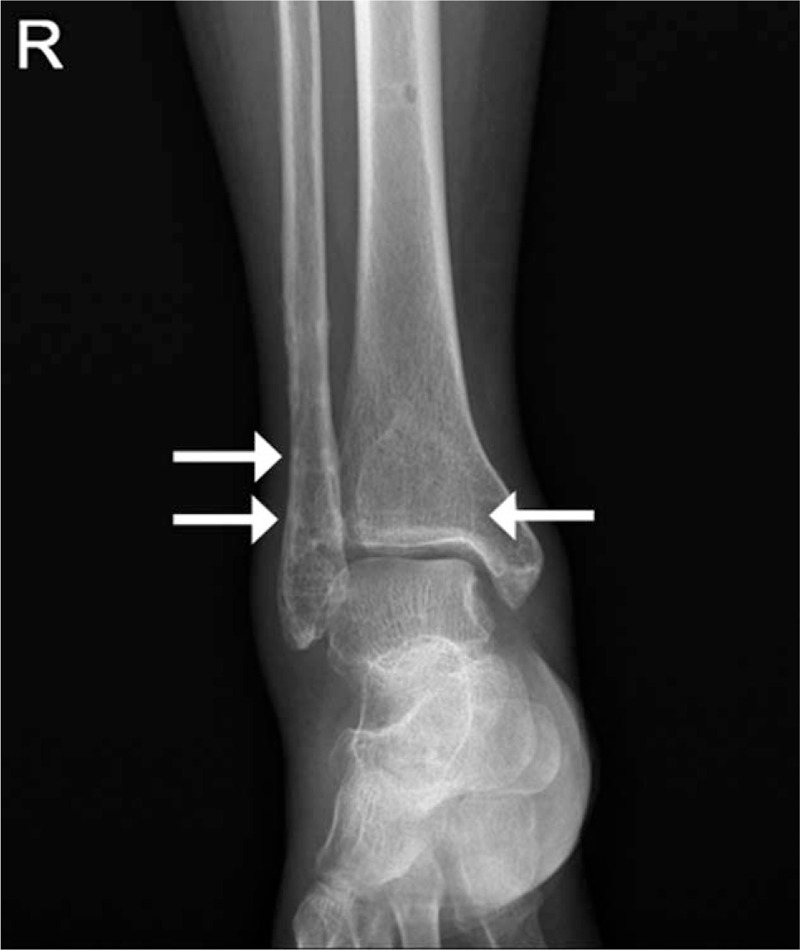
Plain ankle radiographs. Anteroposterior view. Previous operative evidence of along the right tibia and fibula (arrows).

**Figure 2:**
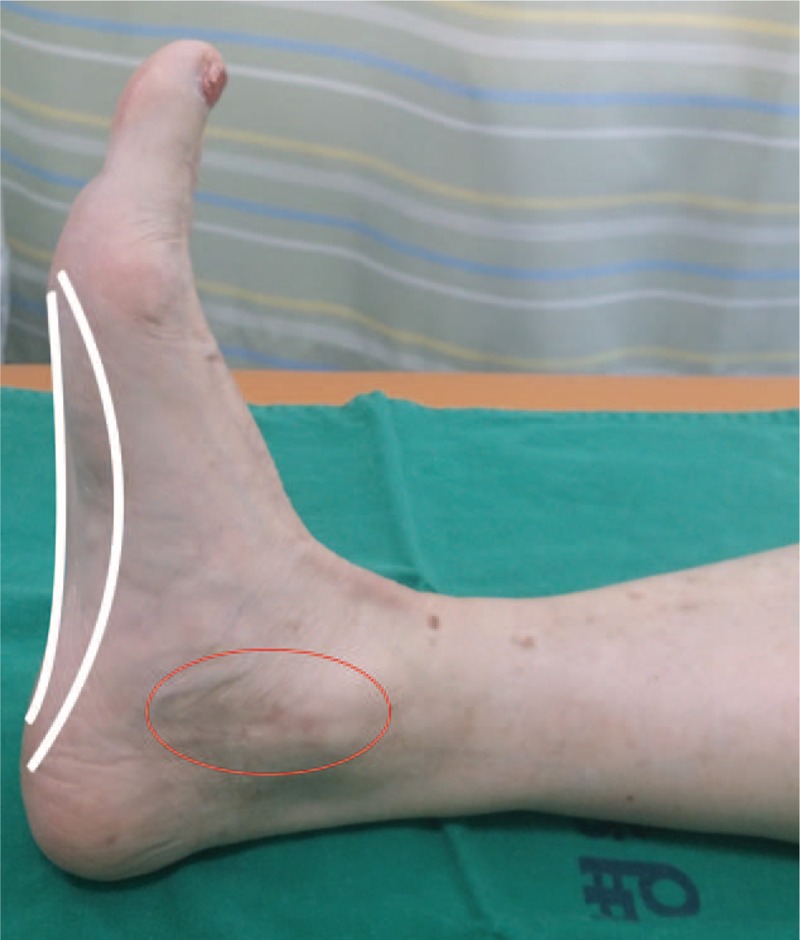
Photograph of the painful location (red circle) and loss of the arch (white line).

**Figure 3:**
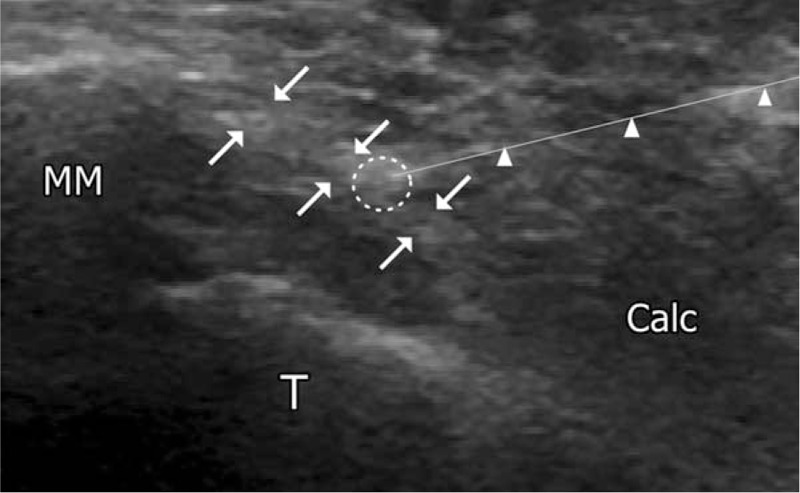
Ultrasound image during injection of polydeoxyribonucleotide in the posterior tibial tendon. Arrowhead indicates the block needle. Calc = calcaneus, posterior tibial tendon (arrows), block needle (arrow head), injection site of polydeoxyribonucleotide (white circle), MM = medial malleolus, T = talus.

A 67-year-old woman presented at our clinic with right ankle pain. The patient measured 153 cm in height, 62 kg in weight, and was overweight with a body mass index of 26.5 kg/m^2^. The patient had undergone syndesmotic screw fixation 3 years prior; however, the pain persisted after the removal of screws from bone (Fig. 1). Additional surgical management was ruled unnecessary by the orthopedic surgeon. She continued to receive physical therapy, such as compression bandages and NSAIDs at the department of orthopedic surgery, for intractable pain of the right ankle, but showed no improvement. She had a history of hypertension that was well controlled with 5 mg amlodipine; in addition, she had suffered from rheumatoid arthritis for 5 years. The patient experienced walking difficulties and pain along the inside of the foot and ankle at the site of the posterior tibial tendon (PTT). Unfortunately, she had suffered from NSAIDs-associated edema for about 1 month due to the adverse effects of long-term orally administered 7.5 mg meloxicam. The orthopedic physician referred the patient to our pain clinic for control of pain and side effect. Meloxicam was discontinued and her pain was managed with fentanyl patch (Matrifen, 12 μg/h transdermal patch, Takeda, UK). Her pain in the medial ankle was described as continuous, crushing, and throbbing and its intensity was registered as 8 on the Numeric Rating Scale (NRS). We reevaluated the problem, taking note of the directionality of her pain, which originated at tibialis posterior muscle and extended to PTT. Physical examination revealed tenderness and swelling on the inside of the ankle, development of a flatfoot, and the loss of the arch; in addition, the tenderness increased particularly during activity (Fig. 2). Based on these findings, the patient was diagnosed as PTTD. Since the patient declined further surgery, prolotherapy with PDRN was considered the treatment of choice. Verbal and written informed consent was obtained from the patient before the procedure. She was instructed to lie in a supine position on the bed and the skin overlying the medial aspect of the ankle was sterilized and prepped. Initially, a superficial skin injection of local anesthetic (1.0% lidocaine) was administered to the injection site. Three or 5 minutes were allowed to pass to ensure effectiveness of the local anesthetic, at which point 3 mL of PDRN was administered into the, tibialis posterior muscle, and PTT under ultrasound guidance (Fig. 3). At the 1 week follow-up after the first PDRN prolotherapy, the NRS score had decreased from 8 to 5, but the patient reported that the ankle was still painful. Therefore, PDRN prolotherapy mixed with 1% lidocaine was delivered into the same site. In the follow-up treatments, the PDRN injection was repeatedly administrated 4 times at 1 week intervals under ultrasound guidance. The patient reported significant pain reduction with decreased NRS scores from 5 to1. The medications were gradually reduced in amount. The patient was followed-up for more than 1 year, and demonstrated good improvement in pain without any complications. At final follow-up, physical examination indicated no swelling and tenderness over the course of the PTT. In addition, she had no weakness inverting (pointing the toes inward) her foot. Patient showed improvement in the arch of the foot, experienced pain relief, and was able to wear regular shoes without any orthopedic device.

## 3 Discussion

We described a case of successful prolotherapy with PDRN in a female patient with PTTD who underwent syndesmotic surgery. PDRN prolotherapy is a safe and effective treatment option and may be considered for PTTD. PTTD is recognized as a leading etiology of acquired flatfoot in middle-aged patients. The primary function of the PTT is to stabilize the hindfoot against eversion and valgus forces. It assists the Achilles tendon in plantar flexion and functions as the main invertor of the foot.^[8]^ The etiology of PTTD is likely to be multifactorial because both extrinsic such as ankle surgery and intrinsic risk factors have been reported.^[9]^ PTTD is more frequent in hypertensive, obese, and rheumatic patients with peak incidence at the age of fifties; in addition, it is 3 times more common in females.^[10]^ In our case, hypertension, rheumatoid arthritis, and overweight were considered as adverse factors in the development of PTTD. PTTD results in structural destabilization of the mid- and hindfoot. Clinically, the continuous deformation of the foot can be classified into 4 stages. In stage 1, the deformity is fully and distinct correctable. In stage 2, the deformity is clear, but still correctable; in stage 3, the deformity becomes stiff. And in stage 4, the ankle is involved in the deformity. Treatment strategies depend on stage. While conservative measures may work in stage 1, surgical treatment is essential for the later stages.^[8]^ However, despite several studies on the clinical effect of treatment modalities, clinical identification of specific stages of PTTD progression remain unclear.^[4]^

Ankle syndesmotic injuries account for up to 25% of ankle sprains^[2]^; and ankle syndesmotic fractures occur in more than 10% of fractures.^[1]^ Syndesmotic injuries are reported to occur less frequently than lateral ankle sprains, however, they occasionally require longer periods of recovery. Syndesmotic injuries can also lead to atrophy, arthrosis and tendon injury.^[11]^ In some cases, satisfactory prognosis can be expected with syndesmotic fixation.^[12]^ However, multiple factors can influence the surgical outcome. Some studies reported long-term functional outcomes for patients with syndesmosis fractures treated with screw fixation and stepwise timed screw removal.^[1]^ Switaj et al^[13]^ reported that unsatisfactory surgical outcome is related to injury factors, surgeon factors, malreduction, hardware-related complications, and patient factors. In our case, the patients’ age was considered to adversely affect surgical outcome; in addition, the main cause of PTTD was attributed to syndesmosis surgery.

She had suffered from NSAIDs-associated edema for about 1 month due to the side effects of orally administered meloxicam. In order to reduce NSAIDs-induced adverse effect, different treatment modalities were considered. A detailed explanation of PDRN was provided to the patient who consented to the treatment strategy. PDRN promotes healing in chronic wounds involving tissue damage by stimulating tissue reconstruction without any adverse effects.^[6]^ PDRN is also known to act on the A2 purinergic receptor selectively to mediate neogenesis and cell growth. PDRN stimulates vascular endothelial growth factor, increases the rate of granulation tissue in fibroblast maturation and differentiation, and consequently accelerates the repair process.^[6,14]^ Moreover, PDRN lowers the expression of the inflammatory cytokines interleukin-6 and tumor necrosis factor—alpha,^[6]^ a potential mechanism for its rapid antiinflammatory effect in PTT. Thus, PDRN was considered an effective therapeutic approach to improve the clinical outcome of PTTD. Chronic and acute toxicity studies confirm that PDRN has no toxic effects on lungs, brain, liver, heart, and skeletal muscle on histologic and macroscopic analysis.^[6]^

Prolotherapy injection is a treatment modality for chronic musculoskeletal diseases. The most commonly used agents are platelet-rich plasma, stem cell, and hypertonic dextrose solution.^[7]^ The presence of a local irritant attracts inflammatory mediators and acts as a vascular sclerosant or stimulates the release of growth factors. Prolotherapy induces a host of inflammatory reactions that result in improved joint function and biomechanics, decreased pain, and stronger connective tissue.^[15]^ Pain relief is possibly related to the elimination of neovessels associated with nerve fibers. Prolotherapy reportedly provides pain reduction for severe refractory Achilles tendinopathy, knee osteoarthritis, chronic plantar fascitis, and lateral epicondylosis.^[7]^ Ultrasound guided prolotherapy improves safety as well as the accuracy for needle placement and ensures that nerve injury is avoided.^[16]^

We effectively treated a female patient with PTTD after syndesmosis surgery using sono guided prolotherapy with PDRN. Future studies are required to ensure PDRN's efficacy. This case indicated that PDRN could positively modify PTTD after ankle syndesmotic injury. It could lower the need for additional medications or therapies and improve the clinical outcomes of patients.

## 4 Informed consent

Informed consent was obtained from the patient for publication of this case report and its related images.
